# “*Armed for the future Coronavirus pandemic*”: a promising use of the multimeric SARS-CoV-2 receptor binding domain nanoparticle as a new Pan-Coronavirus vaccine

**DOI:** 10.1038/s41392-021-00721-1

**Published:** 2021-08-17

**Authors:** Manoj Kumar, Souhaila Al Khodor

**Affiliations:** Research Department, Sidra Medicine, Doha, Qatar

**Keywords:** Infectious diseases, Vaccines

In a recent article in *Nature*, Saunders et al. reported a new pan-coronavirus (CoVs) vaccine that elicited abundant neutralizing antibodies (nAbs) and was proven effective in protecting against a variety of coronavirus infections—including severe acute respiratory syndrome coronavirus-2 (SARS-CoV-2), SARS-CoV-1, and other related bat-CoVs that could potentially cause the next pandemic.^1^

The SARS-CoV-2 caused Coronavirus Disease 2019 (COVID-19) outbreak has posed a severe threat to the global public health. SARS-CoV-2 is a new member of the pathogenic human coronavirus family, which also includes two common mild beta-coronaviruses; hCov-OC43 and hCoV-HKU1, as well as the life-threatening SARS-CoV-1 and MERS-CoV.^2^ Like other beta-CoVs, SARS-CoV-2 harbors a single-strand positive-sense RNA, which encodes nucleocapsid (N), envelop (E), membrane (M), and spike (S) proteins. The S protein contains S1 and S2 subunits. Although the exact etiology of beta-CoVs infection remains elusive, the binding of these viruses to human cell via the receptor binding domain (RBD) of the S1 subunit appears to be the hallmark of their pathogenesis. Numerous studies have reported high titers of nAbs in patients who recovered from SARS-CoV-2 infection, with the majority of the isolated nAbs targeting viral S-protein, particularly the RBD,^3^ highlighting the importance of RBD epitopes in priming the human immune system and mediating protection against SARS-CoV2 infection. As a result, the RBD is a promising target for developing vaccine candidates and boosting the immune system’s ability to combat SARS-CoV-2 infection.

**Fig. 1:**
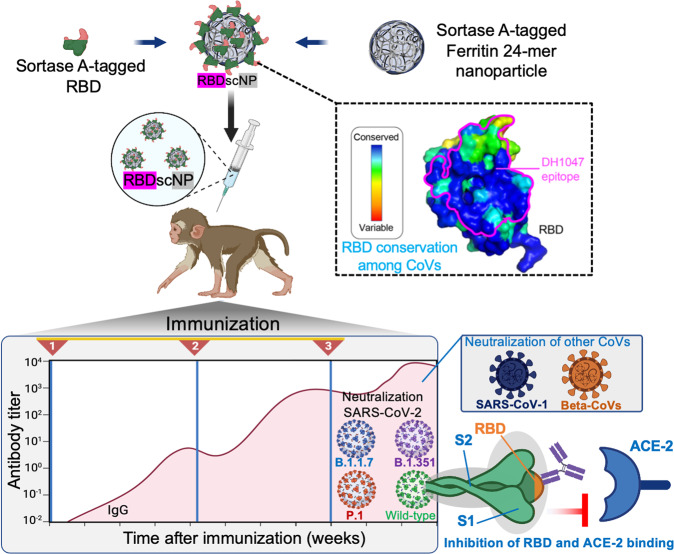
Schematic representation of the key steps followed to develop and evaluate the SARS-scNP vaccine. The RBD-scNP vaccine elicits extremely high titers of neutralizing antibodies (IgG) against SARS-CoV-2 virus, its emerging variants and other related beta-CoVs including BatCoV-WIV-1, and BatCoV-SHC014. RBD harbors conserved epitopes, including the DH1047 epitope within beta-CoV spikes. Receptor binding domain (RBD); angiotensin-converting enzyme-2 (ACE-2); RBD-sortase A conjugated nanoparticle (RBD-scNP); immunoglobulin G (IgG); severe acute respiratory syndrome coronavirus-2 (SARS-CoV-2); coronaviruses (CoVs)

In this report, Saunders et al. designed a novel 24-mer SARS-CoV-2 RBD-sortase A conjugated nanoparticle (RBD-scNP) vaccine.^1^ The recombinant RBD and ferritin nanoparticle were conjugated together by a sortase A reaction and combined with a small molecule adjuvant 3M-052 formulated with Alum, the toll-like receptor 7 and 8 agonists, to boost the immune response. Although, immunization of cynomolgus macaques with the RBD-scNP vaccine elicited high RBD-specific nAbs titers, which significantly neutralized viral infection, the most striking finding was that the neutralizing IgG induced by the RBD-scNP vaccine, not only covers the emerging variants of SARS-CoV-2 but was also able to cross-neutralize other coronaviruses, SARS-CoV-1 and beta-CoVs including BatCoV-WIV-1, and BatCoV-SHC014 with a range of potencies (Fig. 1). This suggests that the highly conserved RBD sequence encodes for a potent antigen capable of eliciting nAbs response that could provide pan-CoVs cross-protection. Competitive binding assays revealed that plasma Abs from RBS-scNP immunized macaques blocked the binding of angiotensin-converting enzyme-2 (ACE-2) and DH1047 nAb to SARS-CoV2, suggesting that RBS-scNP vaccine elicited DH1047-like antibodies. Interestingly, the RBD-scNP vaccine elicited a significantly higher magnitude of DH1047-like antibodies in immunized macaques than both the natural human SARS-CoV-2 infection and the mRNA-lipid nanoparticle (LNP) vaccine, which is similar to the licensed Pfizer/BNT162b2 mRNA-LNP SARS-CoV2 vaccine formulations. This work will pave the way towards developing a pan-CoVs vaccine that can protect from future coronavirus outbreaks.^4^

To further confirm the vaccine protection against natural CoV infection, Saunders et al. also challenged RBD-scNP vaccinated macaques with 10^5^ plaque-forming units of SARS-CoV-2 virus via intratracheal and intranasal routes after their last vaccine boost. Interestingly, the vaccine exhibited a complete protection against SARS-CoV-2 infection as no detectable infectious virus was recovered from the nasal swab and bronchoalveolar lavage (BAL) fluid of vaccinated macaques four days after the challenge. In contrast, as expected, unimmunized macaques showed a high viral load in the range of 10^4–5^ copies/mL in their BAL fluids and nasal swabs. These findings were further supported by the hematoxylin and eosin staining of the lung tissues that showed a reduced inflammation in the immunized macaques compared to the unimmunized macaques infected with the virus.

Taken together, this study by Saunders *el al*. indicates that the RBD-scNP vaccine immunization triggers an ample protective titer of SARS-CoV-2 nAbs and confers a robust protection against SARS-CoV-2 in both upper and lower respiratory tracts. SARS-CoV-2 is a dynamic virus, and it has surprised the scientific world with its fast pace of adaptabilities and mutations. Emerging functional studies have demonstrated the increased infectivity and reduced sensitivity of new SARS-CoV-2 variants, B.1.1.7, B.1.351, and P.1 to some of the current vaccines, due to the mutations in the RBD domain at K417N, E484K and N501Y positions.^5^ In this paper, Saunders et al. showed that three doses of the RBD-scNP vaccine elicited high levels of nAbs to neutralize all variants of SARS-CoV2, and that the binding of those antibodies to SARS-CoV2 was unaffected by mutations observed in B.1.1.7, B.1.351, and P.1 variants.

In summary, things were moving quickly in the last one year of the COVID-19 pandemic, and cutting-edge methodologies are being combined with advanced fundamental procedures by the pharmaceuticals and institutional researchers across the globe to identify new vaccine candidates in order to contain the SARS-CoV-2 pandemic. Interestingly, the mobilization of resources and dedication of scientists has resulted in the identification of more than 300 vaccine candidates in a very short span of time. These vaccine candidates are either in development or currently being used in the battle against the SARS-CoV-2 pandemic. However, as we have experienced three coronavirus epidemics in the last two decades, and bats appear to harbor the different bat-CoVs that could lead to novel human pathogens in the future, this highlights the need for more potent pan-CoV vaccines prior to the next pandemic.

Consistent with this hypothesis, although the pioneering work by Saunders et al. provides a very detailed perspective of the crucial RBD functionality in CoV pathogenesis and high protective efficacies of RBD-scNP immunization to known circulating SARS-CoV2 variants and other bat beta-CoVs, the dose-dependent response of the RBD-scNP vaccine has not been confirmed. T-cell responses, particularly CD8 subsets of CD4 should be assessed after immunization with the RBD-scNP vaccine to ensure activation of virus-specific cellular immune responses. Moreover, because the ferritin 24-mer vaccine core may possibly produce ferritin-specific Abs, it is still important to assess the RBD-scNP vaccine’s potential long-term toxicity to ensure its safety.
